# How Social Media Analysis Offers an Opportunity to Understand the Reality of People Living With Multiple Sclerosis: Descriptive French Study

**DOI:** 10.2196/88763

**Published:** 2026-08-19

**Authors:** Emmanuelle Leray, Stéphane Schück, Pamela Voillot, Nathalie Texier

**Affiliations:** 1 Univ Rennes, EHESP, CNRS, Inserm, ARENES UMR 6051, RSMS U 1309, EHESP, Avenue du Pr Léon Bernard, Rennes, 35033, France, 33 0299022513; 2 Kapcode, Paris, France

**Keywords:** multiple sclerosis, social media, quality of life, burden, testimonies

## Abstract

**Background:**

Multiple sclerosis (MS) is a chronic neurological disease that starts in young adulthood and can significantly affect quality of life (QoL) due to various symptoms, and the risk of disability. MS directly affects people living with the disease and indirectly affects their relatives and family caregivers.

**Objective:**

The objective of this social media analysis was to identify the main topics of discussion among people affected by MS and their perceptions of the impact of MS on their QoL.

**Methods:**

Publicly available French messages, posted between January 2017 and October 2022, were retrieved using an extraction query that contained keywords related to MS. The effects on QoL were detected using a machine learning algorithm specifically trained on social media data. Five specific models covered the following health-related QoL dimensions: physical well-being, psychological well-being, daily activities (including professional and academic activities), social or relational well-being, and material well-being. Descriptive statistics were provided and illustrated with quotes from social media.

**Results:**

The analysis corpus for the 2017 to 2022 period included 3225 messages corresponding to 2034 different social media users, either people living with MS (654/3225, 20%) messages or family caregivers (2571/3225, 80%) messages, identified from 32 sources. Women represented 42.5% (864/2034) and men represented 28.2% (574/2034) of social media users (gender was unknown for 596/2034, 29.3%), and their mean age was 35 (SD 6.6) years. The 2 main themes of posts were “Caregivers and family members” (1032/3225, 32%) and “Disability” (774/3225, 24%). Overall, 847 messages described at least one impact of MS on QoL: relational or social (n=431, 50.9%), physical (n=284, 33.5%), psychological (n=76, 9.0%), financial or material well-being (n=32, 3.8%), and daily activities (n=24, 2.8%).

**Conclusions:**

Our findings confirm the high impact of MS on everyday life and QoL for both patients and family caregivers. Caregivers were the most numerous to express themselves and post messages on social media. The most affected QoL dimension was relational or social well-being, which is probably linked to the fact that social networks and digital patient communities are places for discussion, sharing experiences, and looking for support. These findings confirm that social media is a way for people affected by MS to express themselves and look for support and understanding. They also show that social media provides an opportunity to discover the fears, questions, needs, and thoughts of those affected by the disease, particularly caregivers, who are rarely considered in research studies.

## Introduction

Multiple sclerosis (MS) is a chronic neurological disease that starts in young adulthood (mean age at disease onset: 30 y), with a female-to-male ratio of 2.5 [1]. According to the most recent estimate (2020), 2.8 million people live with MS worldwide [2].

MS symptoms are variable and unpredictable, and in each patient, they can change or fluctuate over time. The most common symptoms are fatigue, walking difficulties, and vision and bladder problems [1]. MS is characterized by physical disability, cognitive impairment, and other symptoms that affect the quality of life (QoL). In addition, as the disease usually begins at a time when people are building their personal lives and careers, it can have a significant impact on their lives and QoL.

Social media has become very popular since the 2000s. Indeed, in 2017, it was estimated that there were over 2.3 billion active social media users worldwide, and this number was growing by approximately 1 million new users every day [3]. In 2025, it was estimated that there would be 5.4 billion social media users worldwide, corresponding to two-thirds of the total global population [4].

The term “social media” refers to internet-based tools that facilitate the gathering of individuals and communities to communicate and share information, ideas, and experiences in real time [3]. Social media have broadly affected medicine, perhaps most publicly by enabling increased communication with and among patients [5]. They allow more rapid, spontaneous, and wider communication and data sharing directly to, from, and between patients. People can anonymously express their feelings and experiences.

To our knowledge, despite the opportunity to access direct, free, and spontaneous testimonies from people living with and/or affected by a disease, few studies in the field of MS have been based on social media analysis. Indeed, we found several analyses of discussions and/or experiences related to specific topics, such as cannabinoid medical use [6], modafinil (a stimulant used in cases of sleep disorders) [7], and pain [8], that included but were not limited to people with MS. Therefore, the aim of the present study was to identify the main themes of discussion by people affected by MS (patients and family/caregivers) on social media and their perceptions of the impact of the disease on their QoL.

## Methods

### Study Design and Population

This noninterventional, retrospective, real-world study included data gathered from social media posts (eg, X, YouTube, Doctissimo, web forums, and blogs, such as la-sclerose-en-plaques.fr and aufeminin.com), written in French by people affected by MS. The term “people affected by MS” refers to people living with the disease, as well as any individual whose life is affected by the disease, such as family members and caregivers. Publicly available messages posted between January 2017 and October 2022 were considered. Private-account posts or data-restricted posts were not accessible and, therefore, were not included.

### Data Extraction

For the present study, an extraction query was constructed (available in Multimedia Appendix 1) with keywords related to MS. All publicly available posts on social media or the web that contained one of the relevant keywords (identified before the extraction) and were posted during the study period were targeted and extracted using the Brandwatch extractor [9]. This tool is based on logical queries using specific keywords evocative of the topic of interest combined with Boolean operators to target specific verbatims. Using the query, the Brandwatch extractor searched through the available sources and posts to find words that matched those in the query. Then, the identified posts were downloaded with metadata (eg, date and web user pseudonym). This constituted the study dataset.

The cleaning process started by removing posts from irrelevant sources, such as potential advertising sites, or messages not fully written in French. The resulting dataset underwent further cleaning to exclusively obtain testimonies by people affected by MS. This was performed using an extreme gradient boosting classifier, a machine learning algorithm that identified posts related to patients’ and caregivers’ experiences with MS. This algorithm was previously trained on a social media dataset constructed using various pathologies and data sources [10,11]. The algorithm used three principal features (syntax, lexical field, and semantic features) to perform predictions. Its recorded performance was as follows: sensitivity of 78% (ie, the proportion of identified true positives) and positive predictive value of 69% (ie, the proportion of true positives among the detected positives). Only posts predicted to have been written by patients or family caregivers were kept, and the others were discarded. Then, a manual review of the predicted posts or authors was performed to exclude false positives. This review was conducted by an expert in semantic analysis of social networks.

The resulting posts constituted the analysis dataset, and the author type (ie, patient or caregiver) was added as a label to each post.

Age and gender were retrieved from information contained in the messages when disclosed by the user (eg, “I’m a 27 yo woman”); otherwise, they were considered to be missing.

In order to highlight the main themes of discussion, the analysis was based on a natural language processing technique (Biterm Topic Models for short text) to automatically identify the topics of discussion addressed in a set of messages. It provides an initial overview of the main topics of discussion. All messages were classified into a single, most representative category based on their content relative to the other messages. A manual interpretation (2 persons) was carried out based on the terms most representative of the category to provide a meaningful title for the category. There is no single metric that can guarantee the best readability or the best clustering of messages. Consequently, several models were tested using different numbers of topics (from 8 to 15), and the most relevant model was then selected based on the maximum value of the log-likelihood, a statistical indicator measuring the extent to which the model correctly explains the observed data. Then, a qualitative manual interpretation phase was carried out to merge certain topics exhibiting a high degree of semantic similarity and to group topics of relatively low importance into an “Other” category.

### Impact of Multiple Sclerosis on Health-Related Quality of Life

The effects of MS on QoL were assessed using a machine learning algorithm specifically trained on social media data [12]. Its operating mode allowed the detection of QoL alterations in verbatims. Then, the effects were classified using 5 specific machine learning models that corresponded to 5 health-related QoL dimensions: physical, psychological, daily activities (including professional and academic activities), social, and material or financial [12]. Then, the output of the models was validated by a health care professional (medical doctor with specific training in data science) to ensure the correctness of the results. This algorithm was previously used in other contexts (atopic dermatitis [13] and oncology treatment [11]).

### Ethical Considerations

By design, this study only included open data publicly available on the web or social media. Private groups, closed online forums, profile information, and personal conversations were not accessed or included. Because of the social platforms’ terms and conditions of use, users give consent for data reuse purposes. This study reused publicly available secondary data without any link to the actual authors of the messages. Furthermore, gathering consent from all users for the retrospective reuse of their data was not technically feasible given the large number of authors.

### Data Analysis

The whole corpus was analyzed using descriptive statistics, and each post corresponded to a statistical unit. The following characteristics were considered: number of posts (overall and by month over the study period), number of authors, discussion themes, occurrence of QoL-related posts and QoL dimensions, data source, and users’ characteristics (gender and age). Examples of posts (verbatim translated from French) are provided to illustrate the findings.

## Results

### Study Population

Over the period 2017 to 2022, the analysis corpus included a total of 3225 messages written in French by 2034 different web or social media users: either people with MS (n=654, 20% messages) or family caregivers (n=2571, 80% messages). They came from 32 different sources.

The respondents’ gender could not be determined for 29.3% (596/2034) of users and was categorized as woman for 42.5% (864/2034) and man for 28.2% (574/2034). Information on age could not be found for 6% (123/2034) of users. The mean age was 35 (SD 6.6) years, and users were predominantly aged 30 to 40 years (57%, 1840/3225 posts).

**Figure 1.**
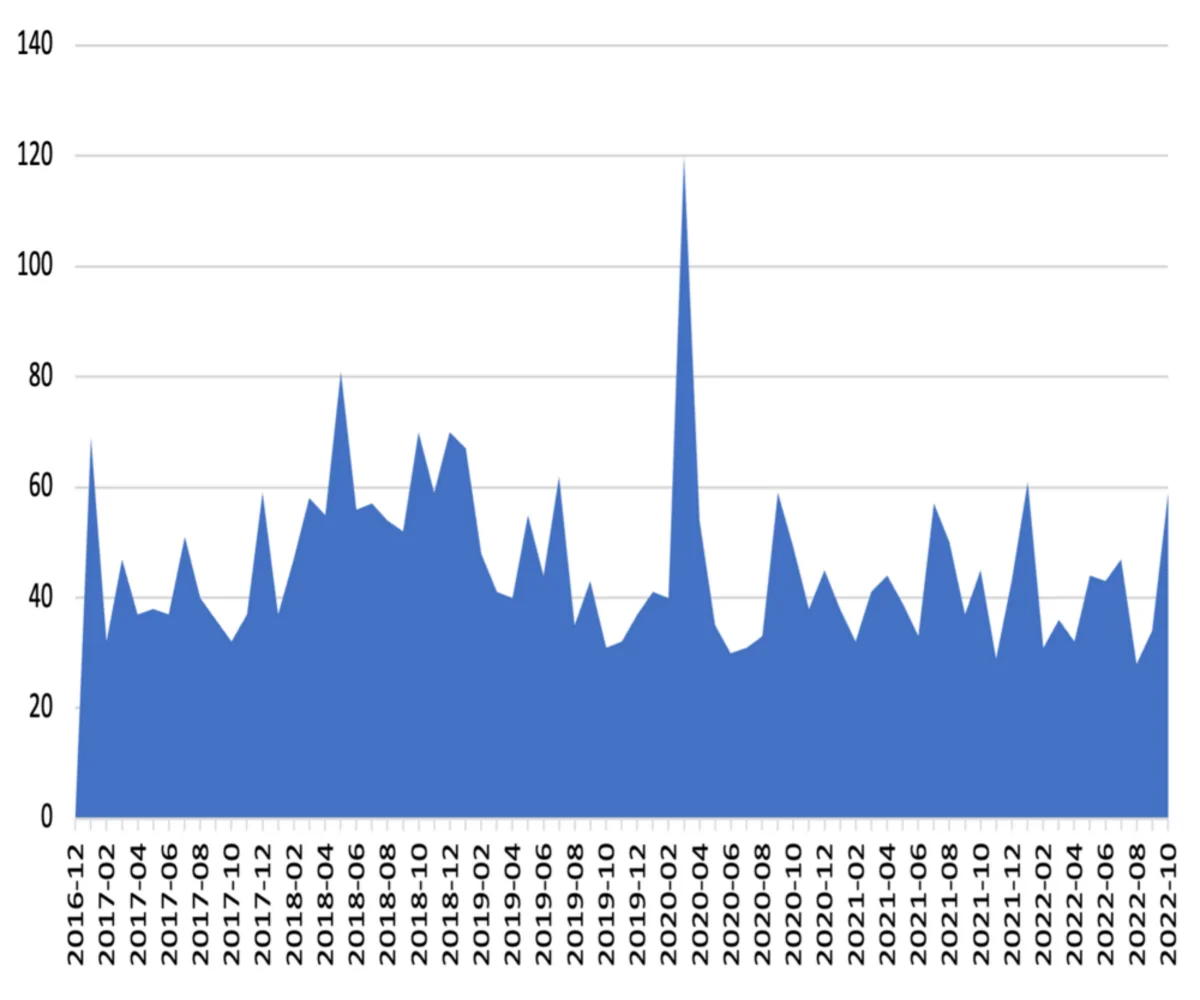
Number of multiple sclerosis–related public posts during the 2017‐2022 period (by month).

Figure 1 shows the monthly number of posts over time. There was no specific trend except some peaks from time to time, notably during World MS Day (May 30 each year) and during the COVID-19 lockdown period that started in March 2020.

### Themes of Discussion

The main themes of the posts are listed in Table 1, overall and according to the patient’s or caregiver’s perspective. The model initially identified an optimal number of 12 themes, determined using the log-likelihood criterion. Subsequently, the manual interpretation phase enabled certain topics with a high degree of semantic similarity to be merged and topics of relatively low importance to be grouped together under an “Other” category, resulting in a total of 7 categories. All the topics related to the disease were covered. People with MS were more likely to talk about their symptoms and the nature of their illness, while caregivers were more likely to talk about themselves and their family members. The 2 most frequent topics are explored below.

**Table 1.** Main themes of discussion identified in the 3225 posts written in French by people affected by multiple sclerosis during the 2017‐2022 period.

Theme	Posts, n (%)	Written by	
		People with MS^a^, %	Caregivers, %
Caregivers and family members	1032 (32)	20	80
Disability	774 (24)	44	56
Symptoms, pain, and fatigue	419 (13)	67	33
Treatments and relapses	323 (10)	69	31
MS impact and difficulties	193 (6)	75	25
Diagnosis, care, and MRI^b^	129 (4)	86	14
Other	355 (11)	—^c^	—

a MS: multiple sclerosis.

b MRI: magnetic resonance imaging.

c Not applicable.

In theme 1, “Caregivers and family members,” which represented 32% (1032/3225) of the corpus, many messages were short and not really informative. Many (2571/3225, 80%) came from family caregivers, and the others (654/3225, 20%) came from people with MS. For instance, children talked about the diagnosis, symptom or disability onset or progression in their mother or father living with MS, or how everyday life was affected by the disease. The way such messages were written suggests the need to share, talk about their experiences, and discuss with people who are going through the same thing.

Examples of posts are as follows:

*At 24, a few months after the birth of my daughter, I found myself with vision loss in one eye caused by a relapse, a diagnosis of #multiple sclerosis and a beautiful baby less than a year old.*

*I received the diagnosis of MS 10 years ago and for the past 6 months I have been feeling really bad (since my second delivery). Headaches, dizziness, neck pain, leg pain and fatigue. I can’t tolerate any treatment, and my morale is starting to take a serious hit!!! The doctors aren’t too concerned, but I am. I really need some support!!*

*I truly understood that my father was ill the day my mother came to pick me up from school and told me that dad could no longer get out of bed because a relapse of #multiple sclerosis had caused him to lose the use of his legs.*

*My mother has multiple sclerosis! It’s been three years now, and it’s been difficult at first, but you have to be strong! Stay strong*

*But my mum has this incredible mental strength. She has multiple sclerosis, there’s no cure, and the only treatment available makes you depressed, but she’s there, dealing with my dad’s moods, her job, my brother...*

Others shared a specific event in their medical history or care pathway to provide feedback to other people and to animate the discussion within the patient community. Few messages evoked parenthood when living with MS or the risk of MS in the family, as exemplified by the following post:

*I spoke with my mother earlier about the fact that the doctors want to check that I don’t have multiple sclerosis (we hadn’t talked about it together yet) and it hurts me because she doesn’t say anything but she’s really worried and she’s not doing well at all, it makes me sad.*

Lastly, a high proportion of messages concerned the risk of COVID-19 and/or vaccination for family members with MS.

Examples of posts are as follows:

*I have multiple sclerosis with immunosuppressive treatment, and I’m one of the most vulnerable people in relation to COVID-19. The start of the new school year on September 1 has me terrified...*

*My mother has multiple sclerosis and is on immunosuppressive treatment. If she catches the virus, well, too bad. I’m starting to get angry that nothing is being done, when it’s clear that it’s spreading.*

Regarding theme 2, “Disability related to MS,” which represented 24% (774/3225) of the posts, many discussions were around “invisible disability.”

Patients and caregivers posted messages saying that the disability associated with MS is not well known or recognized by the general population and also by the administration or social security.

Examples of posts are as follows:

*Thank you very much for this video and for talking about invisible disabilities. My sister has multiple sclerosis and received her diagnosis four months ago, but her illness and above all, the prejudices of her boss, who refuses to make accommodation for her, have forced her to quit. Her boss does not understand the irregular, unpredictable, yet exhausting nature of this condition on a daily basis. I sincerely hope that your video will encourage people who get in contact with individuals with disabilities to reconsider their preconceptions and give them a chance to be more than just a disease.*

*No real handicap! A bit like the big idiots who park in disabled spaces and who don’t think it’s a big deal to take my father’s space because he walks (with a limp). Except that he has multiple sclerosis and it’s not written on his face!*

Some messages reported that disability was the consequence of symptoms in a very personal and qualitative way. These testimonies indicated the important part of pain and fatigue in the disease course. Likewise, many social media users discussed the potential impacts of such symptoms and of disability (eg, activities and work).

Examples of posts are as follows:

*I have an appointment with my neurologist on June 6... I have tingling sensations on my face, hands and legs! It feels like electric shocks in my legs, cramps... I feel like I’m walking drunk, I lose my balance... I won’t even mention the fatigue and pain throughout my body.*

*I have a lot of pain with my multiple sclerosis. I’m in category 1 [Disability pension], but I can’t do my job anymore. Continuous pain and my tiredness mean that I have to force myself to work. I have a lot of pain with my multiple sclerosis, and I have relapses. I’d like to move up to category 2 and be able to stop working, but I don’t know if I need to make an appointment with the social security doctors. And will they understand what I put up with every day? I’m afraid they won’t understand me.*

Some posts were short, and the disability issue could be identified because the word “wheelchair” was used in association with MS.

### Impact of Multiple Sclerosis on Quality of Life

**Figure 2.**
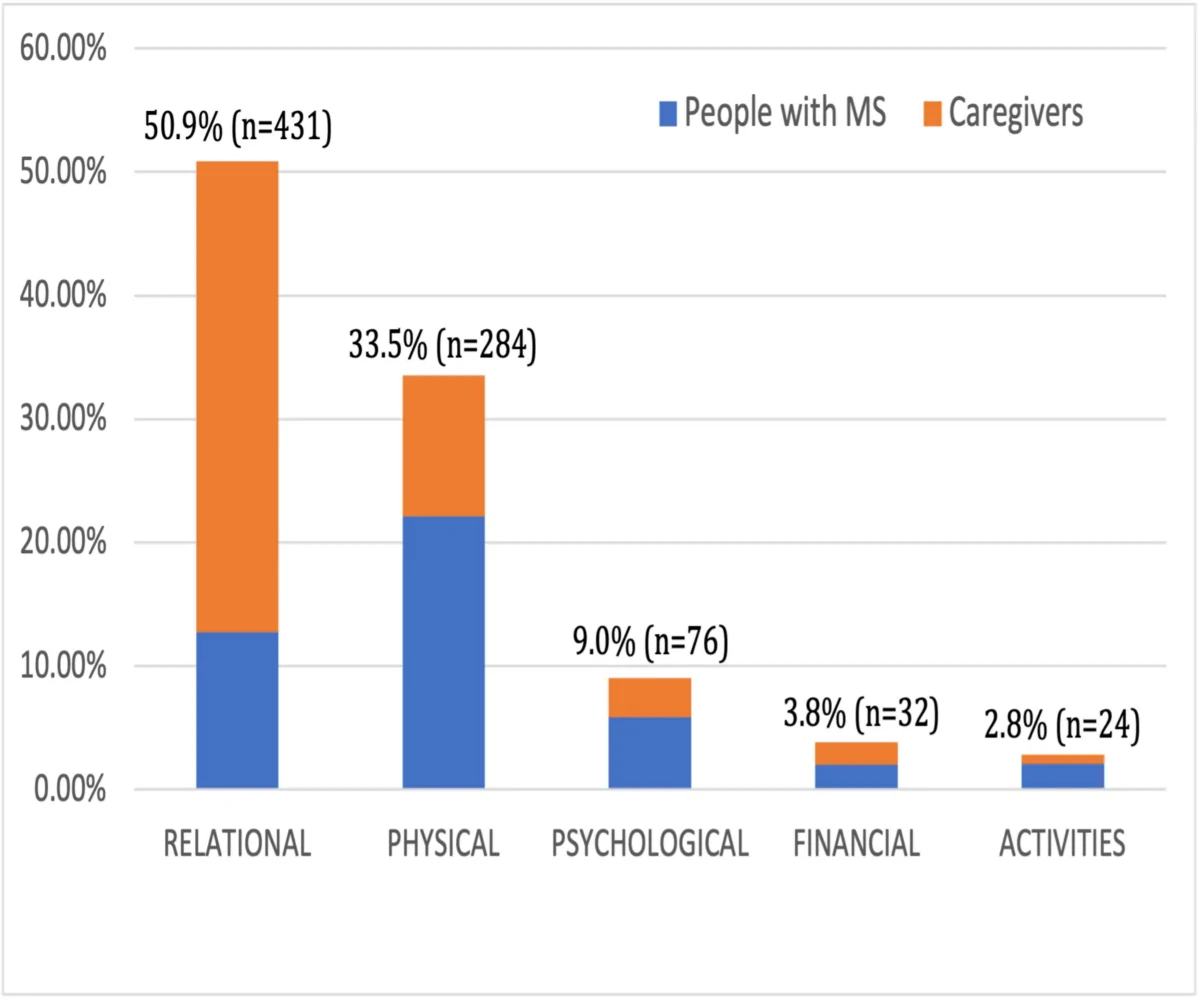
Percentage of public posts concerning the different health-related quality-of-life dimensions (N=847), overall and according to the writers’ status (patient with MS or family caregiver). MS: multiple sclerosis.

Overall, 847 verbatims (ie, 26% of the total corpus) contained at least one impact of MS on QoL (Figure 2). There was no direct correlation between the thematic analysis and QoL analysis, but the results were consistent and logical (main themes: caregivers and disability; main impacts on QoL: relational and physical).

The relational or social impact was the most frequently mentioned (50.9%, 431/847), especially by caregivers. It included effects on social and family life, such as family proximity, social isolation, and sexual relationships. The family aspect was recurrent in the testimonies (73% of posts). For instance, many posts said that the diagnosis of MS extends to the whole family. Some messages talked about unconditional help and support from family members, but also about cases of suffering, neglect, and divorce linked to the disease.

Examples of posts are as follows:

*My sister was great, she helped me to walk again and to get around without rushing me. She made me think about other things. She mentally supported me. I don’t know how to thank her. #carer #multiple sclerosis.*

*I look after my friend all the time and I'm always on the alert, it’s exhausting*.

*I have multiple sclerosis, and I have little doubt about what I will experience in the future: solitude, loneliness, isolation, patience, willingness and so much more.*

*I’ve known from the start that my wife has multiple sclerosis #multiple sclerosis, but that has never stopped me from looking to the future with her, including building a family.*

Patients (4%) who no longer wanted to be a burden on people around them also mentioned isolation.

Examples of posts are as follows:

*My first reaction after the diagnosis #multiple sclerosis (6 years ago) was to divorce to avoid this ordeal for my husband... It was stupid... The two of us are stronger and my husband supports me in an exceptional manner.*

*Simple example: my cousin has multiple sclerosis; she risks to become paralyzed later on, so she doesn’t want to have a child if she’s going to be a burden because of her future disability.*

The physical impact was the second QoL-related topic (284/847, 33.5%) and was most frequently reported by people with MS. It was defined as the impact on the physical condition, such as physical fatigue, motor limitations, pain, and weight gain or loss. In 48% of posts, the authors described various types of pain in their daily lives, including migraine and diffuse pain at the time of relapses.

Examples of posts are as follows:

*I have an appointment with my neurologist on June 6... I have tingling on my face, hands and legs! A sort of electric shock in my legs, cramps... The impression of walking as if I were drunk, loss of balance... I’m not even talking about the fatigue, or the pain all over my body.*

*My father was “placed” in a long-term care facility at the age of 38; his multiple sclerosis had become too much for his wife and mother of his two young children to handle.*

Disability and its progression were mentioned in several testimonies (22% of posts), evoking different types of plegia and/or loss of sensation (visual symptoms, for instance).

An example of a post is as follows:

*I was stubborn, I didn't want to use crutches or walking sticks. As a result, I fell a lot*.

Lastly, 2% of posts concerned the severe fatigue experienced by people living with MS, and its impact on their everyday life.

The psychological impact was the third QoL-related topic (76/847, 9.0%) and was also frequently reported by people with MS. It referred to the effects on the psychological condition, such as moral fatigue, self-image and self-esteem, loneliness, depression, and hope. In 25% of the posts, patients and their family caregivers referred to MS by using the expression “daily struggle.” They underlined the difficulties related to the disease and its symptoms, but also the difficulties related to the management and care of this chronic lifelong disease.

Examples of posts are as follows:

*Because when I received the diagnosis of MS at the age of 40, I had the impression that everything was falling apart, even though days later I was starting the fight.*

*No, I’m not being treated at a university hospital, but at a regular hospital. I didn’t have a hard time accepting my MS, but having 6 relapses in 9 months is just very hard on the body and the mind. At the beginning of my MS, I had two relapses. And before my pregnancy, I had none, although I’ve had MS for 13 years and have taken all the treatments.*

*I have multiple sclerosis, and I have a pretty good idea of what I need to learn in this life: loneliness, isolation, patience, willpower... and many other things besides.*

*I watch over my friend all the time and am always on the alert, it’s exhausting.* #caregiver #multiple sclerosis

Panic, anxiety, and terror toward the future, disease progression, the occurrence of new relapses, or health events could be identified in 15% of the posts:

*Why are you crying, Mum? Each wave of pain reminds me that my multiple sclerosis will probably prevent me from seeing you grow, see you grow up and that I won’t be able to feed myself*.

Lastly, in 6% of the messages, loneliness was discussed by people living with MS, with the feeling of being sidelined by their disease and by society.

The material or financial impact was the fourth QoL topic (32/847, 3.8%). This category included the effect on the financial situation that can lead to debts, bank loans, and difficulties in care management. In this category, 47% of the posts referred to the social security system, including obtaining long-term disease status for MS (“Affection de Longue Durée” in French). In the French health insurance system, individuals with this status have full health care coverage. Messages that mentioned this status reported either satisfaction, requests for information, or discussions on the risk of losing their rights. In several testimonies (34%), patients and their caregivers expressed their need and desire to receive the “Allocation Adulte Handicapé” (allowance for disabled adults) but regretted that the administration refused their application:

*The MDPH [French organism that helps people with disabilities] refused my boyfriend’s #AAH [application]. Saying that his disease is fluctuating. Well, yes, yes. It’s true that his #MS doesn’t tire him out at all, doesn’t make his legs ache, doesn’t give him migraines, and doesn’t prevent him from finding work*.

Lastly, in 22% of the messages, the authors mentioned financial difficulties related to their chronic condition, through budget calculations that underlined the high cost of living. Some shared an online fundraising pot.

An example of a post is as follows:

*I have multiple sclerosis, and I can’t work. So, can you explain to me how, on €860 a month, I can change my car? I can’t take out a loan*.

The impact on daily activities was the least mentioned by people affected by MS and consequently was the fifth and last QoL topic (24/847, 2.8%). In 42% of the posts, people expressed their difficulty in keeping their jobs, particularly when symptoms were increasing.

Examples of posts are as follows:

*Because of multiple sclerosis, I could no longer do my job. I tried to retrain but I couldn’t follow the courses.*

*I’ve got multiple sclerosis and frankly it’s hard to find a job.*

*I lost my old job because of multiple sclerosis, which I’ve had since I was 16...*

*If I’m struggling to feel well today, it’s not because of my MS (...), but because of my employers...*

In 37% of the posts, difficulties in walking, running, and other leisure activities, such as horse riding, were mentioned. Several people also reported the use of a wheelchair, which was seen as a marker of disability.

Examples of posts are as follows:

*Since I’ve had multiple sclerosis, I’ve stopped running. My dog helps me to keep my balance.*

*My daughter is 4. She can see that Mum can’t run behind her like she used to, it’s impossible to take her bike to the park and some days I need crutches. I say that my legs hurt, that the doctor is looking for how to treat me #multiple sclerosis.*

In 8% of the posts, urinary symptoms, such as dysuria, and their consequences were evoked.

## Discussion

### Principal Results

The aim of this infodemiology retrospective study [14] was to describe how French-speaking people affected by MS communicated on social media and web forums about their disease in general and about their QoL in the last 5 years. To this aim, we included 3225 posts from more than 30 sources during the study period. The 2 main themes of posts were “Caregivers and family members” (32%) and “Disability” (24%), which confirms that social media is a way to express the reality and burden of people affected by MS. Most of the messages were written by caregivers (2571/3225, 80%), which probably explains the high frequency of messages relating to relationships and family members. Overall, 847 posts described at least one impact of MS on QoL: relational or social (n=431, 50.9%), physical (n=284, 33.5%), psychological (n=76, 9.0%), and material or financial well-being (n=32, 3.8%), as well as daily activities (n=24, 2.8%). Those results were coherent with the thematic analysis.

Most of the social media users whose messages were analyzed were women aged 30 to 40 years. This corresponds to the age when many people receive a diagnosis of MS and to the age of people who routinely use social media. Among the authors of the retained posts, 42% were women and 28% were men, and 80% (2571/3225) were family caregivers. The large proportion of caregivers in the sample highlights the burden that the disease places on families. We used examples of posts to illustrate our analysis because we think that this is the best way to describe what people are saying on social media.

In the present study, we assessed the impact of MS on QoL through 5 dimensions: physical well-being, social or relational well-being, material or financial well-being, emotional or psychological well-being, and development or activity. The relational impact of the disease was the most frequently mentioned on social media (431/847, 50.9%). This does not mean that the impact of MS on QoL is mainly or only relational. In addition to the fact that many posts were written by caregivers, we think that this is linked to the fact that social networks and digital patient communities are places for discussion, sharing experiences, and looking for support and, therefore, are particularly conducive to capturing this information. Indeed, it is likely that people affected by chronic diseases (patients and family caregivers) experience painful emotions and specific situations in their everyday lives. Caregivers have widely reported the impact on relationships, while people with MS have also reported an impact on their physical and psychological QoL. Posts on social media illustrate their need to share these emotions and experiences and to discuss them with people experiencing the same thing. This is consistent with a recent study on data on the reach and engagement of Facebook posts [15]. This analysis of 5881 comments demonstrated that people with chronic health conditions want to engage on social media and find value in supporting and sharing their experiences with others. This study included people living with MS, migraine, irritable bowel syndrome, rheumatoid arthritis, lung cancer, and prostate cancer.

### Comparison With Prior Work

In the literature, we found 2 studies on posts related to MS, 1 on Twitter [16] and 1 on Facebook [17]. The first one [16] analyzed 74,076 original tweets on MS written in English from February to June 2019 and identified 4 main topics: related chronic conditions, disease burden, disease-modifying drugs, and awareness raising. Its findings were consistent with those of the other study based on 7029 posts from 2 MS Facebook groups [17]. The authors reported 8 information categories: information and awareness (70.0%), event advertising and petitions (5.2%), fundraising (5.0%), patient support (3.1%), drug discussion (2.1%), clinical trials and research studies (0.8%), product and drug advertising (0.7%), and other (13.1%).

Giunti et al [16] also found that tweets on disease burden and related chronic conditions had the most negative feelings, probably linked to their emotional burden, which seems to be in accordance with our results on QoL. In addition, Della Rosa and Sen [17] reported a high engagement level (eg, in terms of views, likes, and comments) for patient support and information or awareness. This confirms our opinion about social media as a means to look for and/or provide support among people affected by MS.

Some posts classified in the “relational or social impact” QoL were not far from the “psychological impact” QoL dimension. Our classification rules may have underestimated this category. Few posts were categorized in the “financial impact” QoL dimension, which may reflect a taboo surrounding this topic when it comes to discussing it publicly. We also found several posts related to the risk of losing a job or not being able to pursue a career. Such fear can affect several impacts of the QoL and reflect the consequences of MS in the everyday lives of young adults.

### Limitations

We recognize some limitations related to our study. First, the analysis concerned only French posts during 5 years, but this does not mean that all people originated from France. Like for all infodemiology studies, only people with internet access who were capable, knowledgeable, and willing to post messages on social media were included. Therefore, this sample might not be representative of the whole population affected by MS [18]. Knowing that patient associations can federate online private communities, it would be of interest to include them in order to better grasp the patients’ reality. Indeed, what patients tell publicly on social media can be different from what they express in private, in patient associations, or with their physicians. Due to ethical and data privacy reasons, our study included only openly accessible online networks and, as a result, could lack potentially relevant information. Nevertheless, social media offers the opportunity to collect data from relatives who share information on the experiences of patients with MS who may not be active on social media.

An extraction bias is also possible because we only considered posts that contained predefined keywords related to our subject. If users expressed themselves by using other words, for instance, without mentioning MS, their posts were not included in the final corpus. To mitigate this bias, the set of keywords was as comprehensive as possible, based on our previous experience in the infodemiology field [8-11].

Another limitation of this study is that multiple posts may originate from a single user. Consequently, certain profiles that are particularly active on social media may be overrepresented in the analyzed corpus, which may influence the apparent frequency of certain themes or reported experiences. Consequently, the results should be interpreted as reflecting the conversations observed online rather than as a representation of the actual prevalence of such experiences within the entire population concerned.

### Conclusion

The present study confirms the high burden of MS and the difficulties in everyday life among people affected by MS. It highlights the burden on family caregivers of people with MS, who were most active on social media in our dataset. Our findings confirm that social media is a way for people affected by MS to express themselves and look for support and understanding. This study also shows that social media provides an opportunity to discover the fears, questions, needs, and thoughts of those affected by the disease, particularly caregivers, who are rarely taken into account in research studies.

## Supplementary material

10.2196/88763 Multimedia Appendix 1 Extraction query in social media.

## Abbreviations

MS: multiple sclerosis
QoL: quality of life
