# Supplementary material for: How Social Media Analysis Offers an Opportunity to Understand the Reality of People Living With Multiple Sclerosis: Descriptive French Study
Source: JMIR Infodemiology. 2026 Aug 19;6:e88763. doi: 10.2196/88763 (PMC13504247; doi:10.2196/88763)
Supplement: Supplementary file 1 [file infodemiology-v6-e88763-s001.docx]

Appendix 1: Extraction query

((SEP OR sclerose OR scerose) NEAR/5 (poussee* OR plaque* OR remittente OR recurrente OR recurente OR recurante OR remitente OR progressive OR primaire))

OR #scleroseenplaque OR #scleroseenplaques OR #BoxonslaSEP OR #journeemondialescleroseenplaques OR #SEPourNous OR #ConnexionSEP OR #VisMaVieAvecLaSEP

OR ocrevus OR ocrelizumab OR ocrelisumab OR lemtrada OR alemtuzumab OR alentuzumab OR teriflunomide OR aubagio OR glatiramere OR tecfidera OR "dimethylfumarate"  OR "dimethilfumarate" OR dimethylfumarate OR copaxon* OR natalizumab OR natalisumab OR tysabri OR tisabri OR mitoxantrone OR elsep OR fingolimod OR glenya OR gilenia OR avonex OR betaferon OR extavia OR rebif OR "interferonbeta"  OR "interferon beta" OR "Criteres de McDonald"  OR (("EDSS") NEAR/5 (score OR echelle OR resultat))

OR ({SEP} NEAR/30 (sante OR medecin OR diagnost* OR maladie OR malade OR traitement OR medicament))
